# Necessity of an Integrative Animal Model for a Comprehensive Study of Attention-Deficit/Hyperactivity Disorder

**DOI:** 10.3390/biomedicines11051260

**Published:** 2023-04-24

**Authors:** Won-Seok Lee, Bo-Eun Yoon

**Affiliations:** Department of Molecular Biology, Dankook University, Cheonan 31116, Chungcheongnam-do, Republic of Korea; wonseoklee@dankook.ac.kr

**Keywords:** ADHD, multifactorial disease, neurodevelopmental disorder, environmental factor, animal model

## Abstract

Animal models of attention-deficit/hyperactivity disorder (ADHD) have been used to study and understand the behavioral, neural, and physiological mechanisms underlying ADHD. These models allow researchers to conduct controlled experiments and manipulate specific brain regions or neurotransmitter systems to investigate the underlying causes of ADHD and test potential drug targets or treatments. However, it is essential to note that while these models can provide valuable insights, they do not ideally mimic the complex and heterogeneous nature of ADHD and should be interpreted cautiously. Additionally, since ADHD is a multifactorial disorder, environmental and epigenetic factors should be considered simultaneously. In this review, the animal models of ADHD reported thus far are classified into genetic, pharmacological, and environmental models, and the limitations of the representative models are discussed. Furthermore, we provide insights into a more reliable alternative model for the comprehensive study of ADHD.

## 1. Introduction

In the early stages of life, the brain and body are still adjusting to the environment [1]. This implies that neurodevelopmental disorders at this stage in life might be cured naturally as children grow or may not be cured, affecting their whole life. The prevalence of attention-deficit/hyperactivity disorder (ADHD) is approximately 10% of children from 4–17 years of age [2]. ADHD is diagnosed in school-aged children, affecting boys more often than girls during childhood and reversing during adulthood. Several studies have shown that approximately 30% of patients develop ADHD during adulthood and later during adolescence. However, even if they recover as adults, suffering from these disabilities during the growth period, when brain development and academic concentration are essential, can be seen as a great loss for individuals and their families [3]. ADHD is characterized by inattention, hyperactivity, and impulsivity. ADHD is a complex neurodevelopmental disorder characterized by disparate behavioral traits and is affected by genetic and environmental factors [4]. For example, ADHD may be caused by intense stress early in life that induces changes in the nervous system [5]. There are also sex differences in the symptoms of ADHD [6]. Several studies have been conducted using animal models. For example, genetic rodent models of a specific gene have been knocked in/out or overexpressed, brain area lesion animal models, and animal models exposed to chemicals or heavy metals, such as polychlorinated biphenyl (PCB), X-rays, or lead [7]. To develop the treatment for ADHD, it is vital to understand the pathological mechanisms, the physiological conditions highly susceptible to the disease, and the disease-related molecular mechanisms. Animal models play pivotal roles in this regard. Therefore, the availability of appropriate animal models is a critical step toward understanding the core mechanisms of ADHD. This review highlights that ADHD is a multifactorial disorder with sex differences and summarizes and critically examines essential information about ADHD-related biological systems and outbreaks, including genetic, pharmacological, and environmental factor models. Therefore, we suggest the necessity of integrative models, such as genetic and environmental models, and performance research that considers heredity, environment, and sex in animal studies.

## 2. Multifactorial Characteristics of ADHD 

Although little is known about the causes of ADHD, it is considered a multifactor model because of the interaction between the genetic causes and environmental factors [8]. According to the results of 20 genetic studies conducted in the United States, Europe, and Australia, the average heritability of ADHD is reported to be as high as 76%. Based on these results, environmental factors are estimated to have an effect of between 20–30% [9]. 

Several animal models and human studies have attempted to elucidate the pathophysiological mechanisms underlying ADHD. Recently, genome-wide association studies (GWAS) and single-nucleotide polymorphism (SNP) studies have been reported [10]. However, single-gene-deficient models have several limitations. In addition, obtaining decisive clues from a GWAS is difficult because various factors have unknown significance. In addition to genetics, some studies have suggested other possible causes and risk factors, including exposure to environmental materials during pregnancy or younger ages. However, observing a clear trend in human research, such as follow-up studies, is difficult. Developing a comprehensive animal model that considers both the environmental and genetic factors is necessary. Therefore, in this study, we investigated the pathological mechanisms underlying ADHD.

## 3. Genetic Model of ADHD

### 3.1. Spontaneously Hypertensive Rats

Spontaneously hypertensive rats (SHRs) have been used as animal models for ADHD, since they were first developed in 1963 [11]. SHRs are bred for high blood pressure and exhibit hyperactivity and impulsivity, similar to the symptoms of ADHD in humans. These rats have been used in several studies to investigate the neurobiological and behavioral mechanisms underlying ADHD and to test the efficacy of various treatments for this disorder over a long period. Generally, when studying this animal model, experiments should be performed before high blood pressure occurs (28 days after birth) to induce hypertension and hyperactivity [12]. SHRs are not perfect representations of human diseases and have been criticized. Nevertheless, SHRs remain one of the most widely used animal models for ADHD and continue to provide valuable insights into the nature of the disorder and its potential treatments. In addition, concerning other factors of ADHD, numerous studies have been conducted in SHR to examine the effects of different drug treatments, such as atomoxetine and methylphenidate, on impulsivity, inattention [13], and hyperactivity [14].

### 3.2. Single Gene Knock-In/Out or Transgenic Mice

Initially, most studies were performed using single-gene knockout mice. Various genetic models that exhibit one or more symptoms of ADHD and alter the expression of the genes related to the neurotransmitter system, such as dopamine, serotonin, and norepinephrine, have been developed. Despite the limitations of genetic models, single-gene knock-in/out or transgenic mice have been considered the best methods to date, because they allow the processing of molecular targets or drugs based on mechanisms related to specific genes and the exploration of pathological mechanisms. In particular, the scientific basis can be reported more reliably because it was based on mechanisms related not only to the catecholamine system (dopamine transporter, dopamine receptor, and a-synuclein), but also to the ADHD-like symptoms with unknown causes, such as the SHR. The characteristics of the representative models are presented in Table 1. 

Dopamine is involved in the control of attention, motivation, and alterations. Therefore, the dopamine system has been implicated in the development of ADHD. Dopamine-transporter knockout (DAT-KO) mice are among the most widely studied ADHD models. Since dopamine cannot be cleared from the synapse due to the lack of dopamine transporters, DAT-KO mice showed hyperactivity. Moreover, DAT-KO mice exhibited hyperactivity by moving greater distances in the open-field test, which could be rescued by treatment with amphetamine, an ADHD drug. Therefore, the DAT-KO mouse model could be used as an ADHD model, showing hyperlocomotion, attention deficits, and impulsivity [33]. Psychostimulants, such as amphetamine, methylphenidate, and cocaine, attenuate hyperactivity in the DAT1-KO model [34]. However, there is no evidence of DAT reduction in patients with human ADHD. Instead, an increase in DAT levels in the striata of adults and children has been observed [35,36]. Dopamine receptors have also been studied for their role in dopamine signaling in ADHD. In particular, the dopamine D4 receptor (Drd4) is associated with ADHD behaviors. In this study, Drd4 knockout mice showed hyperactivity via spontaneous locomotor activity and an open-field test [17]. In a human study, the Drd4 gene in people who showed more ADHD symptoms was found to be altered via SNP analysis. People with changes in the Drd4 gene showed more ADHD symptoms, with higher levels of inattention, hyperactivity, and impulsivity [37]. In a context similar to the dopamine-related theory, the a-synuclein lacking mice proposed as having ADHD was related to the dopamine system [38]. However, this model is excluded from this review because it was used to study Parkinson’s disease and requires further confirmation related to attention deficits and impulsiveness.

Recently, a new single-gene-targeting deletion model has been reported. For example, 14-3-3γ deficient heterozygote mice display hyperactive and depressive-like behavior [39]. However, these are still in the early stages of research, and more studies are needed to fully understand the role of 14-3-3γ in ADHD and its potential as a therapeutic target. Further research is required to confirm these findings and determine the potential therapeutic implications for ADHD.

## 4. Pharmacological Model of ADHD

Pharmacological models that facilitate experiments beyond the variables and limitations of genetic models have also been widely used in ADHD research.

### 4.1. 6-Hydroxydopamine

Dopamine is one of the most widely studied neurotransmitters associated with ADHD. Initially, experimental destruction of DA-containing neurons with 6-hydroxydopamine (6-OHDA) in adult rats was established as a model of Parkinson’s disease. Researchers observed that lesions in the dopaminergic system of neonatal rats lead to age-limited spontaneous motor hyperactivity, relieving these symptoms in adults [40]. Therefore, models injected with 6-OHDA have long been proposed as pharmacological models of ADHD. However, results regarding impulsive behavior and attentional deficits have not yet been reported. 

### 4.2. Nicotine

A prenatal nicotine exposure mouse model has been reported showing hyperactivity and responses to methylphenidate treatment [41]. Additionally, attention and working memory deficits have been reported in this model [42]. This model can be classified as environmental because environmental factors, such as maternal smoking, can affect offspring neurodevelopment and behavior. However, it can be considered a more direct pharmacological model in that nicotine was added to drinking water without using cigarette smoking boxes in the animal models. Maternal smoking during pregnancy affects the pre- and postnatal development of the fetus, increases the risk rate of the fetus, and affects the cognitive and behavioral development in childhood and adolescence [43]. Animal experiments have consistently reported that low weight occurs in cases of prior exposure to prenatal smoking, a well-known risk factor for ADHD. Animal experimental studies have reported that exposure to nicotine before the prenatal phase causes cognitive decline. Smoking inhibits normal placental function and reduces uterine blood flow, leading to hypoxia, ischemia, and malnutrition by reducing fetal nutrients and oxygen and delaying fetal uterine growth [44]. The carbon monoxide and tar in cigarettes directly affect the fetal brain. Nicotine acts directly on the nicotinic receptor of acetylcholine in the fetal brain, causing abnormal cell proliferation and differentiation. In addition, exposure to nicotine before prenatal care resulted in a decrease in the response to dopamine and noradrenaline or low activity. Pre-pregnancy nicotine administration, subsequently, weakened the noradrenaline-induced reaction, which is consistent with the abnormal status of catecholamines in ADHD. Nicotine also affects the dopamine system and persistent prenatal exposure to nicotine in mice damages the structure of the hippocampus [45].

### 4.3. N-Methyl-D-Aspartate Receptor Agonist (MK-801)

Recently, the N-methyl-D-aspartate receptor agonist (MK-801), an agonist of the N-methyl-D-aspartate (NMDA) receptor, was shown to effectively induce various symptoms of different neuropsychiatric disorders in a dose-dependent manner. A sensitivity has been observed against spatial memory impairment and impulsive behaviors at low concentrations of MK-801 [46]. However, some of these symptoms are shared by schizophrenia and autism spectrum disorders (ASD). Although it may be beneficial to investigate NMDA-related mechanisms in ADHD, more in-depth research should be conducted for use as a general ADHD model.

### 4.4. Yohimbine

In non-human primates, α2A-adrenoreceptors in the prefrontal cortex (PFC) are associated with prefrontal cognitive dysfunction in ADHD. Monkeys administered with yohimbine, an α2A-adrenoreceptors antagonist, in the PFC showed impaired no-go performance in the go/no-go task, indicating impulsive behavior [47]. This study implied that α2A-adrenoreceptors in the PFC are involved in the inhibitory control of behavior. Moreover, dysfunction of the locomotor activity was also regulated by a2A-adrenoreceptors in the PFC of non-human primates following yohimbine administration [48]. Therefore, α2A-adrenoreceptors in PFC dysfunction could be factors causing ADHD symptoms, such as impulsive behavior and hyperactivity. However, studies on the physiological changes and molecular mechanisms in this animal model are lacking. Therefore, studies on pathological phenomena and molecular mechanisms are required for models that exhibit ADHD-like behavioral patterns.

## 5. Environmental Model of ADHD

### 5.1. Heavy Metals

Maternal heavy metal exposure is frequently associated with an increased risk of ADHD [49]. Lead is a toxic heavy metal found in the environment, particularly in contaminated water, soil, and old paint. It has been reported that brain development can be significantly affected by relatively low lead concentrations below 10 μg/dL and can lead to cognitive function and behavioral problems [50]. Lead affects several neurotransmitters and is involved in vascular brain capillary integration, synaptic formation, aquatic nation, and catecholamine metabolism in the central nervous system, which affects the dopamine nervous system of the striatum frontal lobe circuit and is associated with the response inhibition and response time variation. When pregnant women are exposed to lead, the metal can cross the placenta and accumulate in the developing fetus. Studies have shown that exposure to lead during pregnancy can have detrimental effects on fetal brain development and increase the risk of behavioral and neurological problems, including ADHD [51]. 

Prenatal cadmium exposure induces behavioral hyperactivity in rat offspring. Therefore, 50 ppm cadmium in the drinking water of pregnant rats was used. Since most heavy metal pollution occurs through water, soil, and air, this review classified it as an environmental factor model. In addition, this effect is enhanced by ethanol co-exposure with cadmium during prenatal development [52].

### 5.2. Polychlorinated Biphenyl

Polychlorinated biphenyls (PCBs) are a group of synthetic chemicals widely used in various industrial and commercial products. PCBs persist in the environment and contaminate food and water. The effects of PCBs on neurodevelopment and the dopamine system have been reported in several cohort studies. As a result, prenatal exposure to PCBs has been reported to result in decreased concentration or focus attention, reduced performance accuracy, and delayed reaction times [53]. The PCB-exposed rat is also a behaviorally well-characterized environmental model of ADHD that reproduces hyperactivity and impulsivity, but does not impair sustained attention. The activities of xenobiotic-metabolizing enzymes in the offspring exposed to PCBs were identified. The PCB-exposed rats exhibited ADHD-like behavior with a short inter-response time in a fixed-interval behavior test, similar to the SHR ADHD model [54]. These studies indicate that PCBs can cause ADHD in humans and rodent models. However, ADHD models induced by PCBs are still under investigation. Therefore, ADHD caused by PCBs needs to be studied further to understand its pathological and molecular mechanisms.

### 5.3. Phthalate

Phthalate is a chemical that makes the plastic used in toys and medical devices flexible and is used as an air freshener. Phthalates have been reported to induce hyperactivity in animal experiments and primarily affect the dopamine system [55]. In addition, phthalates cause abnormalities in the hormonal system and decrease the number of midbrain dopamine neurons [56]. Recently, there have been reports of transgenerational effects on social behavioral abnormalities in offspring exposed to phthalates during pregnancy. Maternal behavior was also observed in this study. Mothers with offspring exposed to phthalate exhibit different maternal behaviors, such as on-nest, liking, and grooming [57]. Social behavioral impairments are representative symptoms of ASD rather than ADHD. The association is still significant, depending on the diversity and heterogeneous characteristics of the symptoms of neurodevelopmental disorders. In addition, environmental factors such as phthalates are not widely used in animal models, but they have been reported to be highly related in cohort studies.

### 5.4. Nitrogen (Hypoxia)

Hypoxia is related to monoamine alterations that further affect neonatal brain lesions. Hypoxia also leads to changes in the release patterns of various neurotransmitters. These factors can lead to the progression of ADHD-like symptoms in animals. ADHD-like behaviors have been reported in nitrogen-induced hypoxic rat models after birth. The treatment period varied depending on age; however, most patients were aged >20 days. These animals exhibited learning and memory deficits and hyperactivity [58]. Morphological changes in the hippocampus are thought to be the main cause of learning deficits [58]. However, it is difficult to assume that this is a single cause and a sequela of hypoxia rather than ADHD.

### 5.5. X-Irradiation

Direct X-ray irradiation of rat pups leads to behavioral deficits. Evidence suggests that a reduction in hippocampal neurons leads to ADHD symptoms [59]. The exposure of rat pups to X-rays results in behavioral defects similar to those observed in human ADHD [60]. In addition, X-rays are considered to be associated with learning and memory deficiencies [61,62]. These X-radiation-induced learning and memory defects were attenuated by amphetamine treatment [59]. However, the practical issues and controversies surrounding this model must be discussed and verified [61].

### 5.6. Stress

The adult offspring of mice treated with restraint stress during pregnancy were hyperactive. Maternally stressed adult mice also show reduced DAT expression and increased DA turnover in the striatum. In addition, treatment with a DA antagonist reduced wheel-running activity. Maternal stress can induce hyperactivity in offspring [63]. An association between ADHD and stress during pregnancy has also been reported in human studies [64,65]. In addition, there is more evidence that offspring born to mothers under sleep deprivation stress show hyperactivity when they become adults. Offspring exposed to maternal stress show increased total time and distance in mobility in the elevated plus-maze test [66]. In addition, it has recently been reported that prenatal exposure to high cortisol levels induces ADHD-like behaviors in rats. Offspring exposed to high cortisol levels showed more hyperactive behavior in the forced swim and open-field tests. Moreover, they spent more time and traveled long distances in the open area in the elevated plus-maze test, indicating higher extremities. These behaviors were consistent with ADHD behavioral symptoms, such as hyperactivity, impulsivity, and inattention [67]. In a human study, individuals exposed to stressful events tended to present with more severe ADHD symptoms. Stressful events include separation or loss, sexual abuse, physical abuse, emotional abuse, domestic violence, emotional and physical neglect, extreme family stress, financial stress and poverty, malnutrition, exposure to heavy metals, other trauma in childhood or adolescence, and prenatal exposure to nicotine [37]. However, further studies are required to investigate the physiological and molecular mechanisms and pathogenesis.

### 5.7. Alcohol (Ethanol)

Drinking during pregnancy is known to cause fetal alcohol syndrome, characterized by mental retardation, abnormal facial features, and a small head size. ADHD and fetal alcohol syndrome differ; however, the behavioral symptoms of fetal alcohol syndrome show patterns similar to those of ADHD. Prenatal alcohol exposure has an overall effect on brain structure, especially in the cerebellum. It has been reported that exposure to alcohol during brain growth acceleration leads to the loss of the cerebellum and Purkinje cells. Extensive studies have been conducted on the effects of alcohol on the brain. In animal studies, cell suicide neurodegeneration increased in alcohol-administered mice, and functional brain magnetic resonance imaging showed difficulty in working memory, a function of the frontal lobe of the abdomen. Single binge consumption has been reported to have neurotoxic effects [68]. Maternal ethanol administration induces ADHD-like behavior in rat offspring. Offspring of the pre-conception ethanol-treated group showed hyperlocomotive activity, attention deficits, and impulsivity. The authors observed a reduction in striatal DAT levels and an increased expression of the norepinephrine transporter in the frontal cortex [69]. In addition, a report that prenatal exposure to ethanol can be used as an animal model of ADHD in rats has been published [70].

## 6. Discussion

ADHD is believed to be caused by genetic, neurological, and environmental factors. Some leading theories suggest that the underlying mechanism of ADHD involves an imbalance in neurotransmitters, such as dopamine and norepinephrine, which regulate attention and behavior. Various drugs have been developed to modulate brain neurotransmitter levels. The most commonly used drugs for ADHD are stimulants, such as methylphenidate and amphetamine. These medications increase the levels of dopamine and norepinephrine in the brain, which can help improve attention and decrease impulsiveness and hyperactivity. In addition to stimulants, non-stimulant medications, such as atomoxetine, are also used to treat ADHD. These medications increase the norepinephrine levels in the brain, which can help improve attention and focus. However, it did not work in approximately 1/3 of the patients. Additionally, although these drugs may be effective treatments for ADHD, the pathology of ADHD remains unknown. Animal models are being developed and used as alternatives to identify the underlying causes of ADHD and effectively treat more patients.

The single-gene knockout homozygote type has been used as an ADHD animal model, but this is a very extreme case in which both copies have been blown away. These homozygous types are favored in animal studies because their clinical symptoms are behaviorally evident. For example, if you look closely at the data in the study that proposed GIT1 knockout as an ADHD model, the knockout type showed a record close to zero in most behavioral experiments. Therefore, it was equal to the control group, but it was impossible to perform such behavioral experiments [23]. This finding is far from the clinical manifestations of ADHD. In the case of the heterozygote type, behavioral symptoms may appear but may not be revealed immediately. It can also appear or disappear in a sex- or age-dependent manner. However, we suggest that these heterozygous types are more reliable models for dealing with genetic and environmental influences.

A combination of environmental factors and genetic variants is known to influence the development and expression of ADHD [37]. This has been studied in the human case and control studies; however, we need to gain insights and apply them in animal models. Although the SHR model of ADHD has been the subject of some debate among researchers, it seems reasonable to investigate the genetic and environmental effects of ADHD using SHR [71]. For example, a cross-species model, or both genetic and environmental factor-induced models, can be used to identify pathological mechanisms and novel therapeutic targets.

The last point that we should not overlook in neurodevelopmental disorders, such as ADHD and ASD, is that these diseases have a large difference in prevalence and symptoms depending on sex. Accordingly, it is necessary to study the molecular and physiological differences that exist prior to pathology. In addition, in clinical practice, drug treatment, and counseling according to sex or symptoms can be appropriately performed. Unfortunately, most previous studies have focused on male mice or rats, as most studies were performed before sex differences in medicine came into the spotlight. Moreover, most researchers have used only male mice in their experiments to achieve ease of study and prevent fluctuations in outcomes that may result from the female menstrual cycle. According to a recent report, only male mice show repetitive behavioral and glial changes in their offspring after exposure to low lead concentrations during pregnancy. The expression patterns of glia-related molecules vary according to gender differences [72]. Therefore, it is necessary to include both males and females in ADHD animal models.

## 7. Conclusions

In this review, animal models of ADHD are classified into genetic, drug, and environmental models. However, the classification of drugs and their environments may be ambiguous. These substances are occasionally administered due to direct exposure to environmental factors. In this study, we focused on the impact of environmental factors on ADHD. In addition to what has been reported thus far, new substances and their effects have been reported. It is estimated that genetic and environmental factors have combined effects, or that more than one environmental factor interacts to cause ADHD. Harmful substances might affect various organs of the body and cause various diseases. Conversely, various substances may interact with one another and cause ADHD. Some hazardous substances may not induce disease, but only cause diseases in people with vulnerable genes. Due to the difficulty of such a complex process, it is necessary to take advantage of multi-factor-induced models or study the interactions between factors rather than only single-factor models in both males and females.

## Figures and Tables

**Table 1:** Single-gene targeting animal model of ADHD.

Model	Mechanism	Representative Feature	Limitation	Others
DAT1-KO	Dopamine transporter 1	-Hyperactivity. -Deficit in learning and memory [15,16]. -Attention deficit in auditory prepulse inhibition (PPI). -Recover by methylphenidate. -Deficit in cliff avoidance reaction (CAR).	Dependency in the background (strain) No clear evidence about dopamine transportation in patients with human ADHD	
Drd4-KO	Dopamine receptor 4	Attention deficit in a 5-choice continuous performance test (5C-CPT). [17]	No deficit in PPI and spontaneous exploratory behavior	
GC-C-KO	Guanylyl cyclase C	Hyperactivity in the home and novel open field. Attention deficit in go/no-go test. Recovered by amphetamine injection (systemic) or GMP-dependent protein kinase agonist (VTA, SNc infusion) [18].	No further study	Selective expression in DA neurons in VTA and SNc
5-HT2C-KO	Serotonin 2c receptor	Impaired 5-choice serial reaction time task (5CSRTT) [19].		
nAchR-KO	Nicotinic acetylcholine receptor	Beta 2: deficit in sustained attention (5CSRTT) [20]. Alpha 5: decrease in accuracy [21]. Alpha 7: attention deficit in 5CSRTT [22].	Phenotypes could be paradigm dependent	
GIT1-KO	G-protein-coupled receptor kinase-interacting protein-1	Exhibit hyperactivity and impaired learning and memory. Enhanced electroencephalography (EEG) theta rhythms. Amphetamine normalizes all phenotypes. At the cellular level, inhibitory transmission but not excitatory transmission is attenuated at GIT1-KO synapses [23].	Controversial report [24]	Association in Brazilian children and adolescents [25]
GAT1-KO	Gamma-aminobutyric acid transporter subtype 1	Hyperactivity, deficits in spatial reference memory. Impaired attentional focusing in an incentive runway test, impulsivity in an incentive passive avoidance test.	[26,27]	
TR-beta 1 transgenic mouse	Carries a mutant human TRb 1 gene	Hyperactivity, impulsivity, inattention. All symptoms are reduced by methylphenidate. Alterations in the dopaminergic system.	[28]	
GFAP-DNSynCAM1	Carrying a dominant-negative form of SynCAM1 specifically targeted to astrocytes	Hyperactivity (disrupted diurnal locomotor activity) with enhanced and more frequent episodes of activity than that of control littermates during the day (when the animals are usually sleeping) accompanied by shorter periods of rest. High levels of basal activity in the dark period (the rodent’s awake/active time) attenuated by amphetamine.	[29]	Specifically targeted to astrocytes
Colombia mutant	SNAP-25 gene	Hyperactivity, impulsivity alterations in the dopaminergic, and noradrenergic systems [30].	Not recovered by methylphenidate [31]	
NF1-KO	Neurofibromatosis type 1	Attention deficits in the lateralized reaction time task. Excitation/inhibition imbalance.	[32]	

## Data Availability

No new data were created.

## Abbreviations

5-choice continuous performance test (5C-CPT), 5-choice serial reaction time task (5CSRTT), 6-hydroxydopamine (6-OHDA), attention-deficit/hyperactivity disorder (ADHD), autism spectrum disorder (ASD), cliff avoidance reaction (CAR), dopamine D4 receptor (Drd4), dopamine transporter (DAT), dopamine transporter knockout (DAT-KO), electroencephalography (EEG), gamma-aminobutyric acid transporter subtype 1 (GAT1), genome-wide association studies (GWAS), G-protein-coupled receptor kinase-interacting protein-1 (GIT1), Guanylyl cyclase C (GC-C), Nicotinic Acetylcholine receptor (nAchR), N-methyl-D-aspartate (NMDA), norepinephrine transporter (NET), phthalate (DEHP), polychlorinated biphenyl (PCB), prefrontal cortex (PFC), pre-pulse inhibition (PPI), serotonin 2c receptor (5-HT2C), single nucleotide polymorphism (SNP), spontaneously hypertensive rats (SHRs).
